# Molecular investigation of *Bartonella* infection among immunocompromised patients in Iran

**DOI:** 10.1016/j.nmni.2026.101770

**Published:** 2026-05-22

**Authors:** Zahra Tahmasebi Ashtiani, Malihe Hasannezhad, Mina Latifian, Ladan Abbasian, Arash Kalantari, Fereshteh Ghaisvand, Mohsen Meidani, Zahra Ahmadinejad, Mohammad Reza Khatami, Fahimeh Bagheri Amiri, Saber Esmaeili

**Affiliations:** a Department of Epidemiology and Biostatistics, Research Centre for Emerging and Reemerging Infectious Diseases, Pasteur Institute of Iran, Tehran, Iran; b National Reference Laboratory of Plague, Tularemia and Q Fever, Research Centre for Emerging and Reemerging Infectious Diseases, Pasteur Institute of Iran, Akanlu, Hamadan, Kabudar Ahang, Iran; c Department of Infectious Diseases and Tropical Medicine, Imam Khomeini Hospital Complex, Tehran University of Medical Sciences, Tehran, Iran; d Iranian Research Center for HIV/AIDS, Imam Khomeini Hospital Complex, Tehran University of Medical Sciences, Tehran, Iran; e Department of Infectious Diseases, School of Medicine, Imam Khomeini Hospital Complex, Tehran University of Medical Sciences, Tehran, Iran; f Department of Immunology and Allergy, Imam Khomeini Hospital Complex, Tehran University of Medical Sciences, Tehran, Iran; g Liver Transplant Research Center, Imam Khomeini Hospital Complex, Tehran University of Medical Sciences, Tehran, Iran; h Department of Infectious Diseases, Imam Khomeini Hospital Complex, Tehran University of Medical Sciences, Tehran, Iran; i Nephrology Research Center, Tehran University of Medical Sciences. the Institution Will Open in a New Tab, Tehran, Iran

**Keywords:** *Bartonella*, Bartonellosis, Immunocompromised, HIV/AIDS

## Abstract

**Background:**

*Bartonella* infections are emerging zoonotic pathogens, primarily transmitted through arthropod vectors or direct contact with animals. Immunocompromised individuals are at increased risk of developing severe complications.

**Methods:**

In this cross-sectional study, a total of 370 blood samples were collected from immunocompromised individuals including patients with HIV, AIDS, common variable immunodeficiency (CVID), liver and kidney transplant recipients, and a control group in Imam Khomeini Hospital in Tehran from May 2024 to February 2025.

**Result:**

Overall, *Bartonella* spp. were detected in 7 out of 370 participants (1.89%). All positive cases were among HIV+ (2.8) and AIDS patients (3.9%). Phylogenetic analysis identified all isolates as *Bartonella quintana*. Univariate analysis suggested that a history of drug abuse and flea infestation seemed to be associated with infection, but due to the rarity of events, multivariate penalized logistic regression did not confirm these as independent risk factors. Cautious interpretation is warranted.

**Conclusion:**

This study provides the first molecular evidence of *Bartonella quintana* infection among immunocompromised patients in Iran, with a notable prevalence observed in HIV-infected individuals, highlighting the need for increased clinical awareness in this population.

## Highlights

• *Bartonella* infection is detected exclusively among HIV-infected individuals, with no cases in other immunocompromised groups or controls.
• All positive samples are identified as *Bartonella quintana* and cluster within the same phylogenetic clade based on 16S rRNA sequencing.
• Illicit drug use, low educational level, and recent ectoparasite infestation are significant risk factors for *Bartonella* infection in HIV-positive patients.

## 1 Introduction

*Bartonella* is a Gram-negative, intracellular, and emerging zoonotic pathogen that can be transmitted through arthropod vectors or via animal bites or scratches. To date, more than 50 *Bartonella* species have been identified in wild and domestic animals, at least 18 of which have been associated with human infections [1]. Notable pathogenic species include *Bartonella henselae*, *Bartonella quintana* and *Bartonella bacilliformis* [2]. Bartonellosis is characterized by intraerythrocytic bacteremia and affects a broad of hosts, including humans, cats, dogs, rodents, and various domestic and wild animals [3]. Multiple arthropod vectors such as fleas, ticks, lice, and sand flies are involved in the transmission of *Bartonella* spp [4].

In humans, bartonellosis often presents with mild symptoms but can occasionally progress to a severe or life-threatening disease. Clinical manifestations may occur in both immunocompromised and immunocompetent individuals even in the absence of identifiable risk factors [5,6]. Severe cases have been associated with complications such as endocarditis, and neuroretinitis [7]. Various *Bartonella* species are responsible for several human diseases, including cat-scratch disease (*B. henselae*), Carrion's disease (*B. bacilliformis*), and trench fever (*B. quintana*). Additionally, both *B. quintana* and *B. henselae* have been implicated in endocarditis, bacillary angiomatosis, and hepatic peliosis [8].

Although *Bartonella* infection occurs in the general population, certain groups remain at substantially higher risk. These include individuals with immunodeficiency disorders, cancer patients, people living with HIV/AIDS, organ transplant recipients, intravenous drug users (IVDUs), homeless individuals, and cat owners [9]. In immunocompromised patients, particularly those with AIDS, *B. henselae* and *B. quintana* may lead to severe complications such as bacillary angiomatosis (BA) and peliosis hepatis. Importantly, *Bartonella* species can persist within erythrocytes for prolonged periods, resulting in chronic or relapsing infections [10]. In 2023, the number of people living with HIV in Iran was estimated at 43,000 (range: 30,000–77,000), of whom only about 24,000 were diagnosed and registered. Among those aware of their HIV status, 71.5% were receiving antiretroviral therapy (ART), and of those on ART who underwent viral load testing, about 92% achieved viral suppression [11].

Bartonellosis in transplant recipients may present with fever and lymphadenopathy making it difficult to distinguish from mycobacterial or fungal infections with similar clinical features [12]. Although treatable, diagnosis remain challenging especially in the absence of specific signs or when symptoms are overlooked. Moreover infection with *B*. *quintana* can manifest with wide range of nonspecific symptoms necessitating careful clinical evaluation to ensure timely diagnosis [13,14].

In Iran, studies on *Bartonella* infections in high-risk populations have primarily focused on patient with culture-negative endocarditis [15,16]. Additionally, the first documented case of bacillary angiomatosis caused by *B. quintana* in an HIV-positive patient in Iran was reported as a case study [17]. Given the lack of comprehensive epidemiological data regarding *Bartonella* infections in immunocompromised populations, this study aims to determine the molecular prevalence and species distribution of bartonellosis among immunocompromised individuals in Iran.

## 2 Methods

### 2.1 Ethical approval and informed consent

This study was approved by the Research Ethics Committee of Pasteur Institute of Iran (IR.PLL.REC.1402.010) and conducted in accordance with principles of the Declaration of Helsinki. All participants’ information was kept strictly confidential and anonymized prior to analysis. Written informed consent was obtained from all patient and controls prior to enrollment.

### 2.2 Study design and population

This cross-sectional study was conducted at Imam Khomeini Hospital from May 2024 to February 2025. Eligible immunocompromised participants were adults (>18 years old) with confirmed immunodeficiency including: I) HIV + group (without AIDS): Individuals with confirmed HIV infection and a CD4 count ≥200 cells/μL, with no history of an AIDS-defining illness, II) AIDS group: Individuals with confirmed HIV infection and a CD4 count <200 cells/μL, III) Patients with common variable immunodeficiency (CVID), and IV) liver and/or kidney transplant recipients. The control group was selected from >18 years old individuals who attended at hospital/laboratory for reasons unrelated to immunodeficiency (e.g., routine checkups or minor surgery). Participants were selected based on clinical records and confirmation of immunocompromised status by a specialist physician. A researcher-made questionnaire was administered to collect demographic and clinical information, including age, sex, city and place of residence, education level, history of drug use, alcohol use, homelessness, animal contact, underlying diseases (diabetic, blood pressure, *Thyroid disorders* and etc), and history of receiving blood and blood products, including plasma-derived products such as intravenous immunoglobulin (IVIG). A 5-ml venous blood sample was collected from each participant and transported under cold chain conditions to the Research Centre for Emerging and Reemerging Infectious Diseases at the Pasteur Institute of Iran for laboratory analysis.

### 2.3 Molecular investigation

Genomic DNA was extracted from all blood samples using a commercial blood DNA Extraction Kit (Viragene Co, Iran) following the manufacturer's instructions. Extracted DNA samples were stored at −20 °C until further testing.

Screening for *Bartonella* DNA was performed using quantitative PCR (qPCR) targeting the *16S–23S rRNA* gene region. [18]. Each 20 -μl reaction mixture included 10 -μl of 2× Real Q Plus Master Mix (Ampliqon, Denmark), 900 nmol each of forward and reverse primers, 200 nmol of probe, and 4 μl of template DNA. The final volume was adjusted using nuclease-free water. Each qPCR run incorporated a no-template control and a positive control containing *B. henselae* DNA to ensure specificity and reliability. Amplification was carried out on the Corbett Rotor-Gene 6000 system (Corbett, Australia) with the following thermal cycling conditions: initial denaturation at 95 °C for 10 min, followed by 45 cycles of 95 °C for 15 s and 60 °C for 60 s. Data analysis was performed using Rotor-Gene® Q software (version 2.3.5; QIAGEN).

### 2.4 Determination of *Bartonella* species

All qPCR-positive samples were further examined for species identification by amplifying the 16S rRNA gene using specific primers [19]. PCR products were separated by electrophoresis on a 1.5% agarose gel. Selected amplicons were purified and submitted for Sanger sequencing (Genomin Co., Iran). = Sequences were analyzed using BLAST against the GenBank database, and species-level identification was assigned based on sequence similarity. Phylogenetic analysis was performed using MEGA version 10.

### 2.5 Statistical analysis

Statistical analyses were performed using IBM SPSS software, version 26.0 and Stata, Version 17Descriptive statistics, including mean with standard deviation for continuous variables, and frequencies with percentages for categorical (including dichotomous) variables, were reported. Univariate regression was used to assess the relationship between *Bartonella* infection and the studied variables. Variables with a univariate p-value <0.25 were considered for the multivariate model. These included: academic education, study groups, cigarette smoking, drug use, exposure to animals, history of receiving blood, and history of flea infestation. Collinearity diagnostics were performed using linear regression on the same set of independent variables. The condition indices were all below 5, and no variance proportions exceeded 0.5 for any pair of predictors (excluding the intercept), indicating that multicollinearity was not a significant issue in our data. The final multivariate model was built using Firth's penalized likelihood logistic regression with all selected variables entered simultaneously. A p-value less than 0.05 was considered statistically significant.

## 3 Results

### 3.1 Demographic and clinical data of participants

A total of 370 individuals were enrolled in this study, including 227 (61.4%) patients with HIV/AIDS (51 patients with AIDS and 176 HIV+), 26 (7.0%) with common variable immunodeficiency (CVID), 57 (15.4%) liver transplant recipients, 13 (3.5%) kidney transplant recipients, and 50 (13.5%) controls. Overall, 203 (54.9%) participants were male and 167 (45.1%) were female. The mean age of participants was 43.56 years ± 13.86 (range 16 to 86 years) (Table 2).

**Table 1:** Oligonucleotide sequences of primer and probe used in this study.

Organism	Gene target	Primer	Sequence (5′- 3′)
*Bartonella spp.*	16S-23S rRNA	Forward	5′-GGGGAAGGTTTTCCGGTTTATC-3′
		Reverse	5′-GAGGACTTGAACCTCCGACC-3′
		Probe	5′-6FAM'-GGAGGGCTTGTAGCTCAGYTGGTTAGAGCG_TAMRA-3′

**Table 2:** The mean and standard deviation of quantitative variables in among immunocompromised patients and control group.

variables	Control n = 51	AIDS N = 51	HIV+ N = 176	Transplant N = 66	CVID N = 26
**Age**	47.1 (14.9)	44.6 (11.3)	45.0 (12.6)	45.2 (14.5)	31.5 (11.7)
**BMI**	26.1 (5.1)	22.5 (5.1)	25.7 (5.2)	25.3 (4.8)	25.5 (18.3)

Among the subgroups, CVID patients had the lowest mean age (31.5 years), while patients with AIDS exhibited the lowest mean BMI (22.5 kg/m^2^). Most participants in all groups resided in urban areas, and approximately 90% of all individuals were residents of Tehran or Alborz provinces. The highest frequencies of smoking, illicit drug use, and alcohol consumption were observed in HIV/AIDS patients (70.6%, 48.0%, and 24.0% respectively) (Table 3).

**Table 3:** The Frequency and percent of frequency for qualitative variables in among immunocompromised patients and control group in Imam Khomeini hospital, Iran (2024-2025).

Variables	Category	N total in each category	Control N = 51	AIDS N = 51	HIV+ N = 176	Transplant N = 66	CVID N = 26
**Sex**	Male	203	19 (37.3%)	38 (74.5%)	99 (56.3%)	33 (50.0%)	14 (53.8%)
	Female	167	32 (62.7%)	13 (25.5%)	77 (43.8%)	33 (50.0%)	12 (46.2%)
**Residence**	Rural	2	0 (0.0%)	0 (0.0%)	1 (0.6%)	1 (1.5%)	0 (0.0%)
	Urban	368	51 (100.0%)	51 (100.0%)	175 (99.4%)	65 (98.5%)	26 (100.0%)
**Province**	Other	44	3 (5.9%)	6 (11.8%)	10 (5.7%)	25 (37.9%)	0 (0.0%)_
	Tehran/Alborz	326	48 (94.1%)	45 (88.2%)	166 (94.3%)	41 (62.1%)	26 (100.0%)
**Years of education**	diploma and lower	138	7 (26.9%)	24 (36.4%)	74 (42.0%)	28 (54.9%)	5 (9.8%)
	Associate degree	139	5 (19.2%)	26 (39.4%)	72 (40.9%)	17 (33.3%)	19 (37.3%)
	Bachelor's degree	64	11 (42.3%)	12 (18.2%)	22 (12.5%)	5 (9.8%)	14 (27.5%)
	Master's degree	25	2 (7.7%)	4 (6.1%)	8 (4.5%)	1 (2.0%)	10 (19.6%)
	Doctorate (PhD/MD)	4	1 (3.8%)	0 (0.0%)	0 (0.0%)	0 (0.0%)	3 (5.9%)
**Smoking**	Yes	129	5 (9.8%)	36 (70.6%)	72 (41.6%)	11 (16.7%)	5 (19.2%)
	No	238	46 (90.2%)	15 (29.4%)	101 (58.4%)	55 (83.3%)	21 (80.8%)
**History of drug abuse**	Yes	56	2 (3.9%)	24 (48.0%)	29 (16.9%)	1 (1.5%)	0 (0.0%)
	No	309	49 (96.1%)	26 (52.0%)	143 (83.1%)	65 (98.5%)	26 (100.0%)
**Alcohol consumption**	Yes	46	5 (10.0%)	12 (24.0%)	24 (13.9%)	2 (3.0%)	3 (11.5%)
	No	319	45 (90.0%)	38 (76.0%)	149 (86.1%)	64 (97.0%)	23 (88.5%)
**Underlying diseases**^a^	Yes	125	14 (27.5%)	16 (31.4%)	33 (18.8%)	53 (80.3%)	9 (34.6%)
	No	245	37 (72.5%)	35 (68.6%)	143 (81.3%)	13 (19.7%)	17 (65.4%)
**Exposure to dogs/cats due to living or working conditions**	Yes	94	5 (9.8%)	22 (43.1%)	49 (27.8%)	11 (16.7%)	7 (26.9%)
	No	276	46 (90.2%)	29 (56.9%)	127 (72.2%)	55 (83.3%)	19 (73.1%)
**Having pet animal**^b^	Yes	80	5 (9.8%)	20 (39.2%)	40 (22.7%)	9 (13.6%)	6 (23.1%)
	No	290	46 (90.2%)	31 (60.8%)	136 (77.3%)	57 (86.4%)	20 (76.9%)
**Exposure to domestic animals**	Yes	18	1 (2.0%)	5 (9.8%)	2 (1.2%)	7 (10.6%)	3 (11.5%)
	No	348	50 (98.0%)	46 (90.2%)	170 (98.8%)	59 (89.4%)	23 (88.5%)
**Cat scratches**	Yes	26	4 (7.8%)	6 (11.8%)	14 (8.0%)	0 (0.0%)	2 (7.7%)
	No	340	47 (92.2%)	45 (88.2%)	158 (89.8%)	66 (100.0%)	24 (92.3%)
**Ectoparasites bite**	Yes	15	1 (2.0%)	5 (10.0%)	5 (2.9%)	1 (1.5%)	3 (12.5%)
	No	346	50.0%)	45 (90.0%)	165 (97.1%)	65 (98.5%)	21 (87.5%)
**Flea infestation in last 5 years**	Yes	2	0.(0.0%)	1 (2.0%)	1 (0.6%)	0 (0.0%)	0 (0.0%)
	No	359	50 (100.0%)	48 (98.0%)	170 (96.6%)	65 (100.0%)	26 (100.0%)
**Rat or Mice Bite/exposure**	Yes	17	0 (0.0%)	6 (12.0%)	4 (2.4%)	2 (3.0%)	5 (20.0%)
	No	344	50 (100.0%)	44 (88.0%)	166 (97.6%)	64 (97.0%)	20 (80.0%)
**Financial income**	No income	137	9 (17.6%)	16 (32.7%)	63 (38.8%)	36 (55.4%)	13 (50.0%)
	<15$	6	0 (0.0%)	2 (4.0%)	4 (2.4%)	0 (0.0%)	0 (0.0%)
	15-75$	22	3 (5.9%)	3 (6.1%)	15 (9.3%)	0 (0.0%)	1 (3.8%)
	75-150$	73	7 (13.7%)	19 (38.7%)	30 (18.5%)	10 (15.4%)	7 (26.9%)
	>150$	115	32 (62.7%)	9 (18.4%)	50 (30.9%)	19 (29.2%)	5 (19.2%)
**Homelessness**	Yes	5	1 (2.0%)	0 (0.0%)	4 (2.4%)	0 (0.0%)	0 (0.0%)
	No	355	50 (98.0%)	50 (100.0%)	164 (97.6%)	65 (100.0%)	26 (100.0%)
Receiving blood	Yes	70	0 (0.0%	14 (27.5%)	26 (14.8%)	28 (42.4%)	2 (7.7%)
	No	300	51(100.0)	37 (72.5%)	150 (85.2%)	38 (57.6%)	24 (92.3%)
receiving plasma-derived products	Yes	106	0 (0.0%)	14 (27.5%)	28 (15.9%)	38 (57.6%)	26 (100.0%)
	No	264	51 (100.0%)	37 (72.5%)	148 (84.1%)	28 (42.4%)	0 (0.0%)

a “Underlying diseases” refers to any chronic non-communicable disease reported by the participant or documented in the medical record. Based on our questionnaire, the following specific conditions were recorded: diabetes type 1 or 2, asthma and allergies, chronic respiratory problems, *Thyroid disorders,* cardiovascular problems, hypertension, history of myocardial infarction or stroke, and dyslipidemia.

b Any pet animal, like cat, dog, birds, etc.

Animal exposure was common: over 40% of patients with AIDS and approximately 30% of HIV-infected individuals reported contact with domestic animals. Pet ownership was also more frequent among patients with AIDS (39.2%). Cat scratches (11.8%) and flea infestation (2.0%) were commonly reported among AIDS patients, while CVID patients had the highest rate of ectoparasite bites (12.5%). Homelessness was reported by four patients in the HIV-positive group (2.4%) and one participant in the control group (2.0%) (Table 3). All CVID patients (100%) reported a history of receiving plasma-derived products, specifically intravenous immunoglobulin (IVIG). Eighty-five percent of all persons had received ART, and the proportion did not differ between positive (85.5%) and negative (85.7%) patients. The mean (SD) viral load in 34 persons was not recorded, but in negative persons (5616.2 ± 51349.1 copies/mL, n = 187) it was higher than in positive persons (119.0 ± 188.6 copies/mL, n = 6); however, the difference was not statistically significant (P = 0.63).

### 3.2 *Bartonella* infections

DNA of *Bartonella* was detected in 7 of 370 participants (1.9%). All positive cases were observed in the HIV/AIDS cohort corresponding to an overall prevalence of 3.1%.No positive cases were identified among the control group. Of these, 5 cases (2.8%) occurred among HIV-positive individuals without AIDS, and 2 cases (3.9%) were detected among patients with AIDS. Education level seemed to be associated with infection: all qPCR-positive individuals had no formal academic education (p = 0.001). Cigarette smokers had a higher odds of *Bartonella* infection (OR 4.76; 95% CI, 0.91–24.88), although this association was not statistically significant. A history of drug using was associated with positivity (OR 7.85; 95% CI, 1.36–71.07). Contact with cats or dogs increased the odds of infection (OR 1.46; 95% CI 0.28–7.68), but this association was not significant. A history of ectoparasite infestation within the past five years were associated with *Bartonella* infection (p = 0.04; OR 58.83; 95% CI, 3.20–1055.30). Flea bites were also more frequent among *Bartonella* positive participants (6.7% vs 1.7%); but the association was not statistically significant (p = 0.20; OR 4.05; 95% CI, 0.46–35.93). No other demographic or clinical variables showed a statistically significant association with *Bartonella* infection (Table 4).

**Table 4:** Univariate logistic regression and penalized multivariate regression analysis of factors associated with Bartonella DNA positivity using qPCR among immunocompromised patients and control group Imam Khomeini hospital, Iran (2024-2025).

Variable	Category	*Bartonella* negative (N = 363)	*Bartonella* positive (N = 7)	Univariate		Multivariate analysis^$^	
		n (%)	n(%)	OR (95%CI)	P value	OR (95%CI)	P value
**Group of study**	HIV+	171 (97.2%)	5 (2.8%)	Reference	0.13	Reference	
	AIDS	49 (96.1%)	2 (3.9%)	1.40 (0.26-7.42)		0.40 (0.06-2.85)	0.36
	CVID	26 (100.0%)	0 (0.0%)	NA		1.55 (0.07-36.14)	0.78
	Transplant	66 (100.0%)	0 (0.0%)	NA		0.21 (0.01-4.44)	0.31
	Control	51 (100.0%)	0 (0.0%)	NA		1.19(0.05-28.02)	0.91
**Age**	<40	143 (98.6%)	2 (1.4%)	Reference	0.71	Not entered in model	
	≥40	220 (97.8%)	5 (2.5%)	1.63 (0.31-8.49)			
**BMI (kg/m^2^)**	<18.5	33 (97.1%)	1 (2.9%)	Reference	0.41	Not entered in model	
	18.5-24.99	163 (98.8%)	2 (1.2%)	0.41 (0.04-4.59)			
	25-29.99	114 (98.3%)	2 (1.7%)	0.58 (0.05-6.59)			
	>30	53 (96.4%)	2 (3.6%)	1.24 (0.11-14.28)			
**Gender**	Male	198 (97.5%)	5 (2.5%)	Reference	0.46	Not entered in model	
	Female	165 (98.8%)	2 (1.2%)	0.48 (0.09-2.51)			
**Place**	Rural	2 (100.0%)	0 (0.0%)	NA	0.99	Not entered in model	
	Urban	361 (98.1%)	7 (1.9%)				
**Province**	Other	43 (97.7%)	1 (2.3%)	Reference	0.99	Not entered in model	
	Tehran/Alborz	320 (98.2%)	6 (1.8%)	0.81 (0.09-6.86)			
**level of Education**	Diploma and lower	131 (94.9%)	7 (5.1%)	NA	0.001	Reference	0.06
	Academic degree	232 (100.0%)	0 (0.0%)			0.07(0.01-1.13)	
**Smoking**	No	236 (99.2%)	2 (0.8%)	Reference	0.06	Reference	0.83
	Yes	124 (96.1%)	5 (3.9%)	4.76 (0.91-24.88)		1.02 (0.11-9.34)	
**Drug use**	No	306 (99.0%)	3 (1.0%)	Reference	0.01	Reference	0.52
	Yes	52 (92.9%)	4 (7.1%)	7.85 (1.71-36.07)		2.31 (0.29-18.18)	
**Alcohol consumption**	No	314 (98.4%)	5 (1.6%)	Reference	0.22	Reference	0.70
	Yes	44 (95.7%)	2 (4.3%)	2.86 (0.54-15.16)		0.50 (0.05-4.64)	
**Underlying diseases**^a^	No	240 (98.0%)	5 (2.0%)	Reference	0.99	Not entered in model	
	Yes	123 (98.4%)	2 (1.6%)	0.78 (0.15-4.08)			
**Exposure to dog/cat**	No	273 (98.9%)	3 (1.1%)	Reference	0.07	Reference	0.26
	Yes	90 (95.7%)	4 (4.3%)	4.04 (0.89-18.41)		3.22 (0.55-18.70)	
**Having pet animal**^b^	No	285 (98.3%)	5 (1.7%)	Reference	0.65	Not entered in model	
	Yes	78 (97.5%)	2 (2.5%)	1.46 (0.28-7.68)			
**Exposure with farm animal**	No	341 (98.0%)	7 (2.0%)	NA	0.99	Not entered in model	
	Yes	18 (100.0%)	0 (0.0%)				
**Cat scratches**	No	340 (98.3%)	6 (1.7%)	Reference	0.26	Not entered in model	
	Yes	14 (93.3%)	1 (6.7%)	4.05 (0.46-35.93)			
**History of flea infestation in last 5 year**	No	353 (98.3%)	6 (1.7%)	Reference	0.04	Reference	0.15
	Yes	1 (50.0%)	1 (50.0%)	58.83 (3.2-1055.30)		38.78 (0.26-5721.81)	
**History of Flea bite in last years**	No	340 (98.3%)	6 (1.7%)	Reference	0.2	Reference	0.75
	Yes	14 (93.3%)	1 (6.7%)	4.05 (0.46-35.93)		0.57 (0.02-17.45)	
**Rat or Mice**	No	337 (98.0%)	7 (2.0%)	NA	0.99	Not entered in model	
	Yes	17 (100.0%)	0 (0.0%)				
**Homelessness**	No	348 (98.0%)	7 (2.0%)	NA	0.99	Not entered in model	
	Yes	5 (100.0%)	0 (0.0%)				
Receiving blood	Yes	67 (95.7%	3 (4.3%)	3.31 (0.73-15.15)	0.13		
	No	296 (98.7%)	4 (1.3%)			2.91 (0.53-16.04)	0.22
receiving plasma-derived products	Yes	104 (98.1%)	2 (1.9%)	0.99 (0.19-5.22)	0.99		
	No	259 (98.1%)	5 (1.9%)				

Frequency (n) and percent of frequency in each category (%), Odds ratio and 95% confidence interval and P value are reported for each analysis.

$ Variables with p > 0.25 in univariate analysis were included.

a “Underlying diseases” refers to any chronic non-communicable disease reported by the participant or documented in the medical record. Based on our questionnaire, the following specific conditions were recorded: diabetes type 1 or 2, asthma and allergies, chronic respiratory problems, *Thyroid disorders,* cardiovascular problems, hypertension, history of myocardial infarction or stroke, and dyslipidemia.

b Any pet animal, like cat, dog, birds, etc.

Multivariable Firth logistic regression revealed no statistically significant predictors of Bartonella infection (overall model p = 0.46). Although academic education showed a borderline protective effect (OR = 0.06, 95% CI: 0.01–1.12, p = 0.06), and flea infestation had a large but imprecise odds ratio (OR = 38.78, 95% CI: 0.26–5721.81.24, p = 0.15), none reached the 0.05 significance level (Table 4).

### 3.3 Determining of *Bartonella* species

**Fig. 1:**
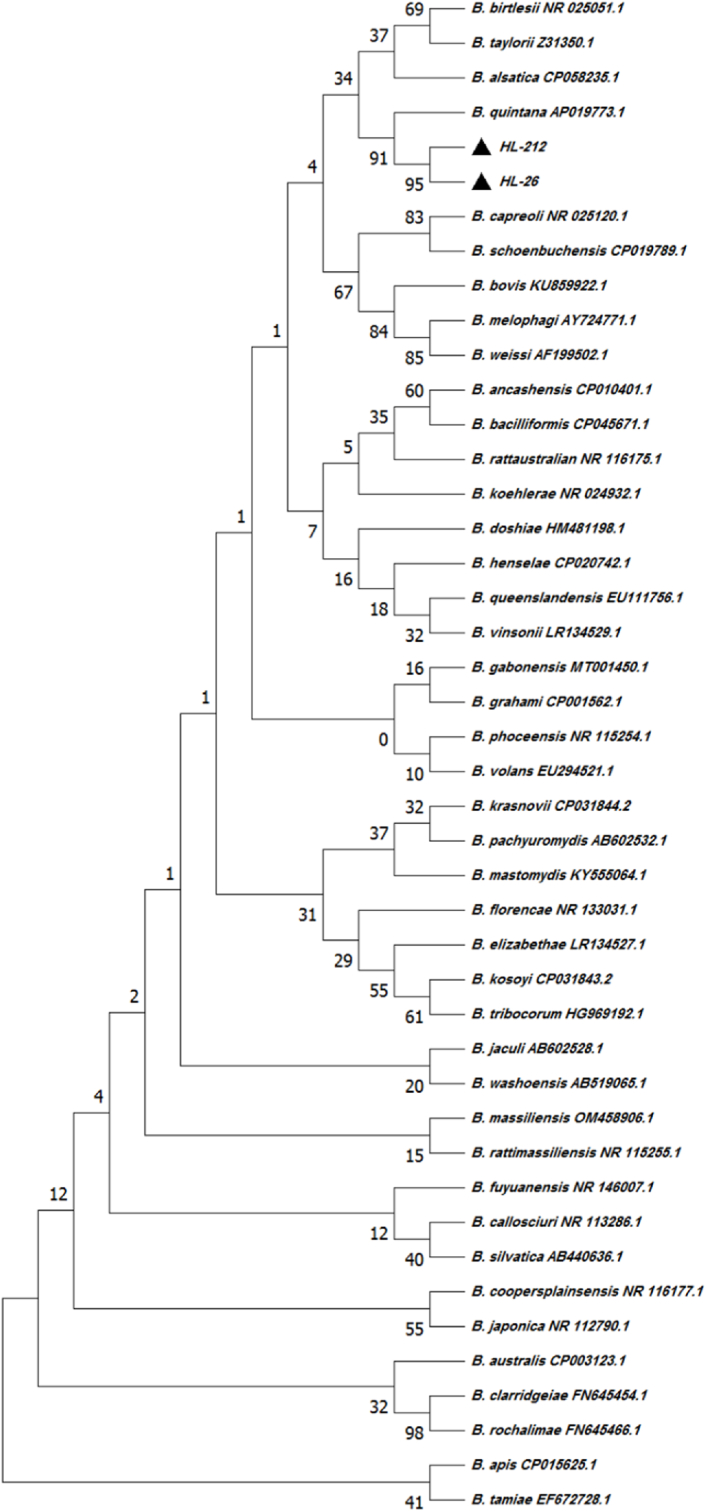
**Bioinformatics analysis of sequences obtained in this study and data related to *Bartonella* species in the GenBank.** The analysis was performed using MEGA software and the Neighbor-Joining method. *Bartonella* spp. identified in this study are indicated by black triangles.

Sequencing analysis of the 16S rRNA gene confirmed that all qPCR-positive samples belonged to *B. quintana*. BLAST analysis demonstrated a high degree of genetic similarity between all isolates and reference strains of *B. quintana*. Notably, two samples (EL-26 and E−2121) showed 100% sequence identity with the *B*. *quintana* reference strain AP019773.1. Phylogenetic analysis further supported the clustering of all isolates within the *B. quintana* clade (Fig. 1; Table 1).

## 4 Discussion

To the best of our knowledge, this study is the first to investigate the prevalence of Bartonella infection among immunocompromised individuals in Iran. Among 370 participants, Bartonella DNA was detected in 1.9%, that all of whom were HIV infected, highlighted a prevalence of 3.1% in this subgroup. Although the number of positive cases was small and did not allow for statistical comparison, the slightly higher prevalence observed among patients with AIDS (3.9%) may reflect the greater degree of immune dysfunction associated with advanced HIV infection. Immunodeficiency due to AIDS could increase susceptibility to opportunistic pathogens such as Bartonella spp. This restriction to the HIV-infected population underscores a clinical concern, as immunocompromised individuals, particularly those with impaired cellular immunity, are at increased risk of opportunistic infections such as bartonellosis [20]. The study conducted in San Francisco evaluated 382 febrile patients and identified Bartonella infection in 18% of cases using culture, indirect fluorescent antibody (IFA) testing, or PCR. *B. henselae* or *B. quintana* was isolated or detected by PCR in 12 patients (3%), all of whom were HIV-positive.These results emphasize that Bartonella infection is a relevant and underrecognized cause of febrile illness among people living with HIV(20). This similarity reinforces the role of Bartonella as an opportunistic pathogen in HIV-positive populations. Bartonella spp. are globally distributed zoonotic pathogens [21] and their clinical severity is often modulated by the host's immune status. As noted in prior literature, Bartonella infections tend to present more severely in immunocompromised individuals, particularly in those with HIV/AIDS or undergoing immunosuppressive therapy [22,23]. The importance of host immunity is further underscored by the fact that Bartonella infection can remain subclinical in immunocompetent individuals but can lead to disseminated disease in those with impaired immune responses.

Although a history of flea infestation within the past five years seemed as statistically significant association with Bartonella DNA positivity, the extremely wide confidence interval indicates substantial uncertainty regarding the true magnitude of this effect. This imprecision is due to the very small number of participants with flea infestation (n = 2), and the estimated odds ratio should therefore be interpreted with caution. While the association is likely genuine, the precise strength of the risk cannot be reliably determined from the current data. Bartonella is primarily transmitted by arthropod vectors, such as fleas, and through contact with domestic animals. Our results may emphasizes the critical role of ectoparasites in the epidemiology of Bartonella transmission [24]. Additionally, a history of drug using seemed associated with infection (p = 0.01), likely reflecting behavioral and socioeconomic vulnerabilities that increase exposure risk. Although the route of drug use is not ascertained, and recognizing prior evidence that subclinical Bartonella bacteremia has been detected among blood donors—raising concerns about transfusion transmission, the hypothesis of Bartonella transmission via injection practices or contaminated blood merits further investigation. Therefore, additional studies are warranted to provide stronger evidence [25]. None of the variables reached statistical significance at the α = 0.05 level in multivariate penalized regression. This is likely due to the small number of positive cases (n = 7), which results in wide confidence intervals and low power to detect associations.

A study from Korea assessed *B. henselae* seroprevalence in healthy adults and reported an overall positivity rate of 15%. The prevalence was significantly higher among individuals who had raised cats (22.2%) compared to those without cat exposure (13.7%). In our study, no statistically significant association was found between exposure to dogs or cats and Bartonella infection. This contrasts with the Korean study in healthy adults, potentially due to differences in host immune status, intensity of exposure, or diagnostic methods [26]. A comprehensive understanding of the intricate interactions between Bartonella spp. its vectors, and its reservoirs, as well as the global distribution of Bartonella spp. infections, is essential for evaluating the public health impact of bartonellosis [27].

The prevalence of *B. quintana* in United States was reported 15% among individuals with a history of homelessness, confirming that *B. quintana* remains a public health concern in the United States and is likely underrecognized based on currently available data [28]. Another study from the United States reported cases of *B. quintana* infection in organ transplant recipients who had received organs from donors with a history of homelessness. This suggests that homelessness may be associated with an increased risk of Bartonella transmission through organ transplantation a finding not observed in our study. The study highlights the potential for transmission via transplanted organs, particularly when donors belong to high-risk populations, such as individuals experiencing homelessness, who are more likely to be exposed to ectoparasites like lice and fleas known vectors of *B. quintana* [9]. While homelessness was uncommon in our study (reported by only four HIV-positive participants and one control), it is important to note that homelessness, drug user, and low socioeconomic status often coexist and may act as indirect factors associated with infection.

Given these risks, *B. quintana* could be considered for classification as a notifiable disease, with initial surveillance efforts focusing on high-risk populations [29]. Given a reported case-fatality approaching 10% [30] *B. quintana* infection requires early diagnosis, ideally before the onset of endocarditis and a high index of suspicion in both at risk individuals and patients without known risk factors, particularly in regions where cases have been reported [6].

Taken together, these findings underscore the need for targeted surveillance of Bartonella spp. in high-risk immunocompromised populations, integration of molecular and serological diagnostic tools, and enhanced clinical awareness, particularly in regions where the infection remains largely under recognized.

### 4.1 Study limitations

In our study wide confidence intervals reflect small numbers; estimates should be interpreted conservatively. One of the limitations of this study is that due to the limited number of immunocompromised patients, sampling was restricted to a single hospital in central Tehran. In some cases, sampling was not possible due to the use of specific medications or isolation requirements. Additionally, the study focused exclusively on the detection of Bartonella spp. in immunocompromised individuals and did not examine potential transmission routes, such as animal contact, tick bites, or exposure to fleas, in detail. Another limitation of this study is the potential for delayed diagnosis, which may lead to the administration of immunosuppressive agents to manage clinical symptoms. This could lead to false-negative PCR results and an underestimation of the true prevalence of Bartonella infection in this population.

## 5 Conclusion

According to the results of our study, *B. quintana* infection was detected among immunocompromised patients at Imam Khomeini Hospital in Tehran, with all positive cases occurring in HIV-infected individuals. Although the overall prevalence was low, the findings underscore the clinical significance of Bartonella infection in high-risk populations, particularly those with impaired immune function. The detection of *B. quintana* in this setting highlights the need for increased clinical awareness and the inclusion of Bartonella in the differential diagnosis of immunocompromised patients.

## CRediT authorship contribution statement

**Zahra Tahmasebi Ashtiani:** Data curation, Investigation, Supervision, Writing – original draft, Writing – review & editing. **Malihe Hasannezhad:** Data curation, Resources, Software, Supervision, Validation. **Mina Latifian:** Data curation, Investigation, Writing – review & editing. **Ladan Abbasian:** Conceptualization, Data curation, Supervision, Visualization. **Arash Kalantari:** Data curation, Investigation, Methodology, Software. **Fereshteh Ghaisvand:** Data curation, Funding acquisition, Investigation, Resources. **Mohsen Meidani:** Resources, Software, Supervision, Validation. **Zahra Ahmadinejad:** Investigation, Software, Validation, Visualization. **Mohammad Reza Khatami:** Software, Supervision, Validation, Visualization, Writing – review & editing. **Fahimeh Bagheri Amiri:** Formal analysis, Funding acquisition, Methodology, Project administration, Resources, Software, Supervision, Validation, Visualization, Writing – review & editing. **Saber Esmaeili:** Conceptualization, Resources, Software, Supervision, Validation, Writing – review & editing.

## Declaration of competing interest

The authors declare that they have no known competing financial interests or personal relationships that could have appeared to influence the work reported in this paper.
